# Enhancement of Adeno-Associated Virus-Mediated Gene Therapy Using Hydroxychloroquine in Murine and Human Tissues

**DOI:** 10.1016/j.omtm.2019.05.012

**Published:** 2019-06-04

**Authors:** Laurel C. Chandler, Alun R. Barnard, Sarah L. Caddy, Maria I. Patrício, Michelle E. McClements, Howell Fu, Cristina Rada, Robert E. MacLaren, Kanmin Xue

**Affiliations:** 1 Nuffield Laboratory of Ophthalmology, Nuffield Department of Clinical Neurosciences, University of Oxford, John Radcliffe Hospital, Oxford OX3 9DU, UK; 2 Oxford Eye Hospital, Oxford University Hospitals NHS Foundation Trust, NIHR Biomedical Research Centre, Oxford OX3 9DU, UK; 3 Medical Research Council Laboratory of Molecular Biology, Cambridge CB2 0QH, UK

**Keywords:** adeno-associated virus, AAV, gene therapy, hydroxychloroquine, chloroquine, innate immunity, Toll-like receptor 9, TLR9, cGAS, APOBEC

## Abstract

The therapeutic effects of gene therapy using adeno-associated virus (AAV) vectors are dependent on the efficacy of viral transduction. Currently, we have reached the safe limits of AAV vector dose, beyond which damaging inflammatory responses are seen. To improve the efficacy of AAV transduction, we treated mouse embryonic fibroblasts, primate retinal pigment epithelial cells, and human retinal explants with hydroxychloroquine (HCQ) 1 h prior to transduction with an AAV2 vector encoding GFP driven by a ubiquitous CAG promoter. This led to a consistent increase in GFP expression, up to 3-fold, compared with vector alone. Comparing subretinal injections of AAV2.CAG.GFP vector alone versus co-injection with 18.75 μM HCQ in paired eyes in mice, mean GFP expression was 4.6-fold higher in retinae co-treated with HCQ without retinal toxicity. A comparative 5.9-fold effect was seen with an AAV8(Y733F).GRK1.GFP vector containing the photoreceptor-specific rhodopsin kinase promoter. While the mechanism of action remains to be fully elucidated, our data suggest that a single pulse of adjunctive HCQ could safely improve AAV transduction *in vivo*, thus providing a novel strategy for enhancing the clinical effects of gene therapy.

## Introduction

Gene therapies based on adeno-associated virus (AAV) vector-mediated gene transfer are at advanced stages of clinical development to treat a broad range of monogenic disorders, including inherited retinal dystrophies,^1^, ^2^, ^3^, ^4^ hemophilia B,^5^ hemophilia A,^6^ muscular dystrophies,^7^ and inherited neurodegenerations.^8^ The approval of voretigene neparvovec by the US Food and Drug Administration (FDA) for the treatment of Leber congenital amaurosis caused by mutations in *RPE65* has paved the way for similar AAV-based gene therapies in the near future.^9^ Recombinant AAV vectors are considered ideal vehicles for transgene delivery compared with lentiviral or adenoviral vectors due to their low immunogenicity; broad tissue tropism; and the ability of the delivered therapeutic transgenes to persist as episomes, which carry low mutagenic potential while allowing sustained transgene expression.

The clinical efficacy of AAV-mediated gene therapy is strongly dependent on the proportion of target cells transduced, particularly when trying to halt disease progression in post-mitotic tissues such as the retinal pigment epithelium (RPE), photoreceptors, neurones, and myocytes. While transduction can be increased to some extent by vector dosage, host inflammatory and immune responses to AAV become limiting factors at high doses, and they may compromise the persistence of transgene expression and therapeutic effects.^10^, ^11^, ^12^, ^13^ Consequently, despite promising results seen with retinal gene therapy, cases of intraocular inflammation have been encountered.^14^, ^15^, ^16^ Therefore, one of the major challenges of gene therapy for retinal diseases is how to achieve sufficient levels of gene replacement at a safe vector dose.

After initially detecting specific upregulation of a panel of intracellular innate immune factors following AAV retinal gene therapy in mice, we investigated hydroxychloroquine (HCQ), a putative inhibitor of anti-viral pattern recognition receptors Toll-like receptor 9 (TLR9) and cyclic guanosine monophosphate (GMP)-AMP synthase (cGAS),^17^, ^18^, ^19^ as a means of improving the efficacy of AAV gene therapy. Here we demonstrate the enhancement of AAV transduction *in vitro*, *ex vivo*, and *in vivo* using HCQ in both murine and human tissues and specifically within RPE and photoreceptor cells. We investigated the effect of HCQ across two commonly used serotypes of AAV, and we assessed its safety and efficacy when co-administered subretinally with AAV *in vivo*. Together, these findings suggest that the adjunctive use of HCQ during AAV gene therapy may provide a safe means of improving AAV transduction within the retina *in vivo*.

## Results

### AAV Retinal Gene Therapy Activates Intracellular Innate Immune Responses *In Vivo*

To assess the activation of innate immune responses to AAV following retinal gene therapy, we first performed subretinal injections of an AAV serotype 2 vector encoding GFP driven by a ubiquitous CAG promoter (AAV2.CAG.GFP) and sham injections of diluent in paired eyes of 4-week-old female C57BL/6J mice. The purity of the AAV vector was confirmed during the production process (Figure S1).

**Figure 1:**
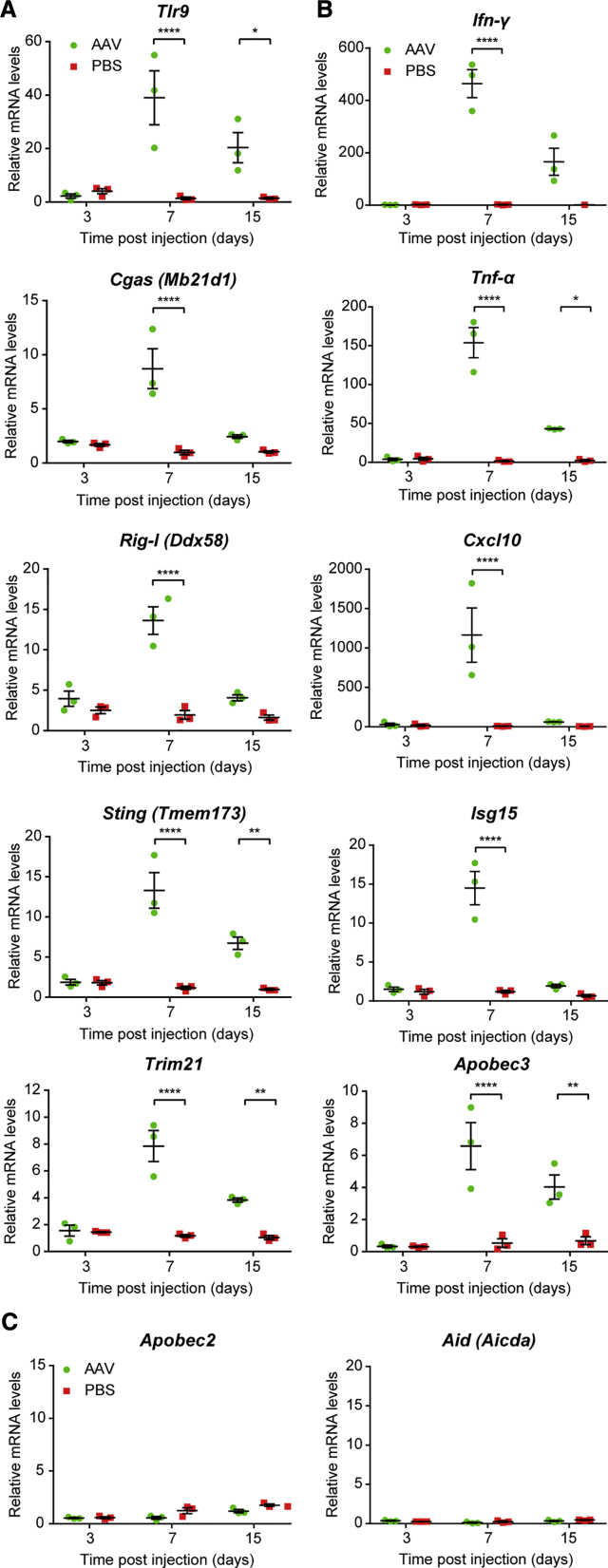
Intracellular Innate Immune Responses Are Activated by AAV Gene Therapy AAV2.GFP vector (1 × 10^9^ gc) was subretinally injected into wild-type C57BL/6 mice, with sham injections of PBS undertaken in the contralateral eye. RNA was extracted from the mouse retina on days 3, 7, and 15 post-injection (n = 3/time point). The expressions of a range of cytosolic anti-viral (A) sensor and (B) effector genes and (C) two genes from the APOBEC family were quantified using qRT-PCR. Relative expression was calculated as a mean fold change (±SEM) relative to the mean of both eyes from uninjected baseline controls (n = 2). *p ≤ 0.05, **p ≤ 0.01, ****p ≤ 0.0001 (two-way ANOVA with Šídák’s multiple comparison test).

Expression of a panel of key anti-viral sensors and effectors (Table 1) was assessed using qRT-PCR of retinal samples collected on days 3, 7, and 15 post-injection. Significant upregulation of the intracellular viral sensors *Tlr9*, *Rig-I* (also known as *Ddx58*), *Cgas* (*Mb21d1*), *Sting* (*Tmem173*), and *Trim21* (Figure 1A) and anti-viral effectors *Ifn-γ*, *Tnf-α*, *Cxcl10*, *Isg1*, and *Apobec3* (Figure 1B) was detected in eyes treated with AAV compared to those sham injected with PBS. Although there was no effect on gene expression soon after gene therapy (3 days post-injection), after 7 days all the viral sensors and effectors were significantly upregulated compared to sham controls, with *Ifn-γ*, *Tnf-α*, and *Cxcl10* exhibiting >10-fold increases in gene expression. By day 15, the expression levels of all these genes dropped relative to day 7, with *Rig-I*, *Mb21d1*, *Ifn-γ*, *Cxcl10*, and *Isg15* no longer demonstrating a significant difference from the sham eye. In contrast, no change in the expressions of *Apobec2* and *Aid* (also known as *Aicda*), both members of the same family of cytidine deaminases as *Apobec3*, was seen over the 15-day course, suggesting that AAV induced selective activation of innate immune responses (Figure 1C). GFP transgene expression was significantly above baseline from day 7 onward (one-way ANOVA, p = 0.0003; n = 5–7) (Figure S2).

**Table 1:** Summary of the Anti-viral Functions of Key Intracellular Innate Immune Mediators

Gene	Alias	Summary of Function	Reference
*Tlr9*		detects unmethylated CpG DNA within the cytosol to activate downstream anti-viral responses	^24^
*Rig-I*	*Ddx58*	detects cytosolic viral RNA, including sequences that have been transcribed by RNA polymerase III from AT-rich dsDNA	^37^
*Cgas*	*Mb21d1*	detects cytosolic dsDNA and synthesizes the secondary signaling molecule cGAMP	38, 39
*Sting*	*Tmem173*	activated by cGAMP, stimulates TBK1 to phosphorylate IRF3, which can induce type I interferon response	40, 41
*Trim21*		detects and neutralizes antibody-bound virions after endosomal escape	42, 43
*Ifn-γ*		type II interferon that controls innate and adaptive anti-viral immune responses	^44^
*Tnf-α*		proinflammatory cytokine that can induce the breakdown of the blood-retina barrier	25, 26, 27
*Cxcl10*		chemokine secreted in response to IFN-γ and activates leukocytes to regulate immune responses	^45^
*Isg15*		an ubiquitin-like molecule stimulated by type I interferon in response to viral infection; covalent and non-covalent binding can disrupt viral replication	^46^
*Apobec3*		deaminates cytidine to uridine in viral DNA, causing hypermutations that inhibit viral replication	^47^
*Apobec2*		lacks identifiable cytidine deaminase activity and no known anti-viral activity	^48^
*Aid*	*Aicda*	cytidine deaminase involved in immunoglobulin gene hypermutation and class switching	^49^

*Tlr9*, Toll-like receptor 9; *Ddx58*, DExD/H-Box Helicase 58; *Rig-I*, retinoic acid-inducible gene 1; *Mb21d1*, mab-21 domain containing 1; Cgas, cyclic GMP-AMP synthase; dsDNA, double-stranded DNA; cGAMP, cyclic GMP-AMP; *Tmem173*, transmembrane protein 173; *Sting*, stimulator of interferon genes; TBK1, TANK-binding kinase 1; IRF3, interferon regulatory factor 3; *Trim21*, tripartite motif containing 21; *Tnf-α*, tumor necrosis factor-α; *Ifn-γ*, interferon-γ; *Cxcl10*, C-X-C motif chemokine ligand 10; *Isg15*, interferon-stimulated gene 15; *Apobec3*, apolipoprotein B mRNA-editing enzyme, catalytic polypeptide 3; *Aicda*, activation-induced cytidine deaminase (*Aid*).

### The Antimalarial Drugs HCQ and Chloroquine Can Increase AAV Transduction in Mouse Embryonic Fibroblasts

Since the activation of proinflammatory cytokines could create an anti-viral environment within the target tissue, thus restricting AAV transgene expression and persistence, we postulated that the suppression of intracellular immunity might enhance the efficacy of AAV transduction. We identified HCQ as a putative inhibitor of two key sentinels in the initiation of intracellular anti-DNA viral response: cGAS, which detects the presence of cytosolic double-stranded DNA (dsDNA),^17^ such as inverted terminal repeats (ITRs) that form secondary structures in single-stranded AAV genomes; and TLR9, which detects unmethylated CpG sequences,^18^, ^20^ also present in AAV ITRs.

**Figure 2:**
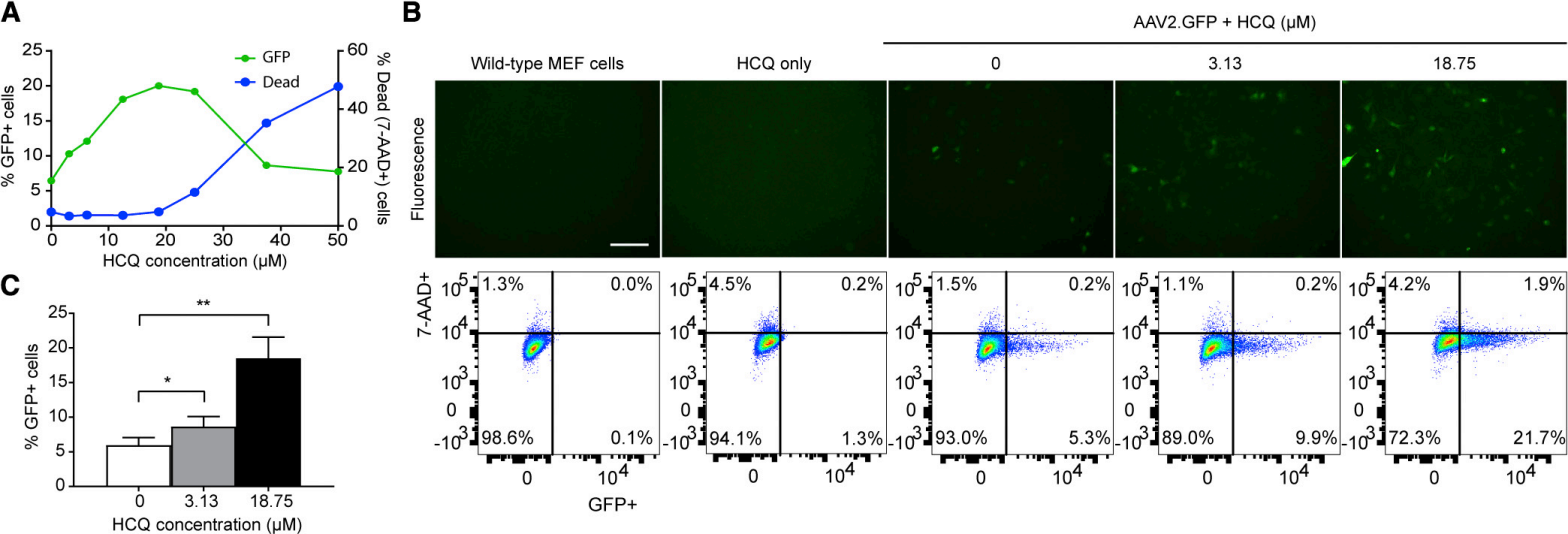
Hydroxychloroquine (HCQ) Increases AAV Transduction in Mouse Embryonic Fibroblasts (MEFs) Wild-type MEFs were pre-treated with HCQ for 1 h prior to transduction with AAV2.GFP at an MOI of 1,000. (A) Representative dose-response curve of HCQ concentration versus either GFP-positive or dead (7-AAD-positive) cells, represented as a percentage of the total number of cells. (B) Representative fluorescence microscopy images of MEFs treated with 0, 3.13, or 18.75 μM HCQ acquired at 3 days post-transduction (scale bar: 200 μm), shown alongside flow cytometry analyses gated for GFP fluorescence and the cell viability marker 7-AAD. (C) Proportion of GFP-positive (GFP+) cells expressed as a percentage of the total number of live (7-AAD-negative) cells at day 3. Data are presented as mean ± SEM (n = 6). *p ≤ 0.05, **p ≤ 0.01 (one-way repeated-measures ANOVA with Dunnett’s multiple comparison test).

In an initial dose-escalation trial, wild-type mouse embryonic fibroblasts (MEFs) were treated with increasing concentrations of HCQ up to 50 μM for 1 h prior to transduction with AAV2.GFP, followed by flow cytometry analysis of GFP fluorescence on day 3. HCQ treatment increased the number of GFP-positive cells up to 18.75 μM; however, concentrations greater than 18.75 μM appeared to be cytotoxic, as evidenced by an increase in 7-aminoactinomycin D (7-AAD) staining, which coincided with a decrease in GFP positivity (Figure 2A). Subsequent in-depth testing of HCQ was limited to 3.13- and 18.75-μM concentrations. The mean proportion of GFP-positive cells increased in a dose-dependent manner from 6% with no HCQ to 9% with 3.13 μM HCQ and 19% with 18.75 μM HCQ (one-way ANOVA, p = 0.0026; n = 6), without adverse effects on cell survival (Figures 2B and 2C).

To see if the enhancement of AAV transduction by HCQ might be a drug class effect, we also tested the structurally similar 4-aminoquinoline antimalarial compound chloroquine (CQ). Pre-incubation of MEFs with CQ for 1 h prior to transduction with AAV2.GFP also improved transduction from 2.5% with no CQ to 6.4% with 12 μM CQ (Figure S3). Since HCQ is known to be less retinal toxic than CQ in patients on long-term therapy for lupus and rheumatoid arthritis,^21^ we chose 3.13 and 18.75 μM HCQ as the minimum and maximum effective concentrations of HCQ to move forward for testing in other *in vitro*, *ex vivo*, and *in vivo* systems.

### HCQ Increases AAV Transduction in Non-human Primate RPE Cells and Human Retina *Ex Vivo*

**Figure 3:**
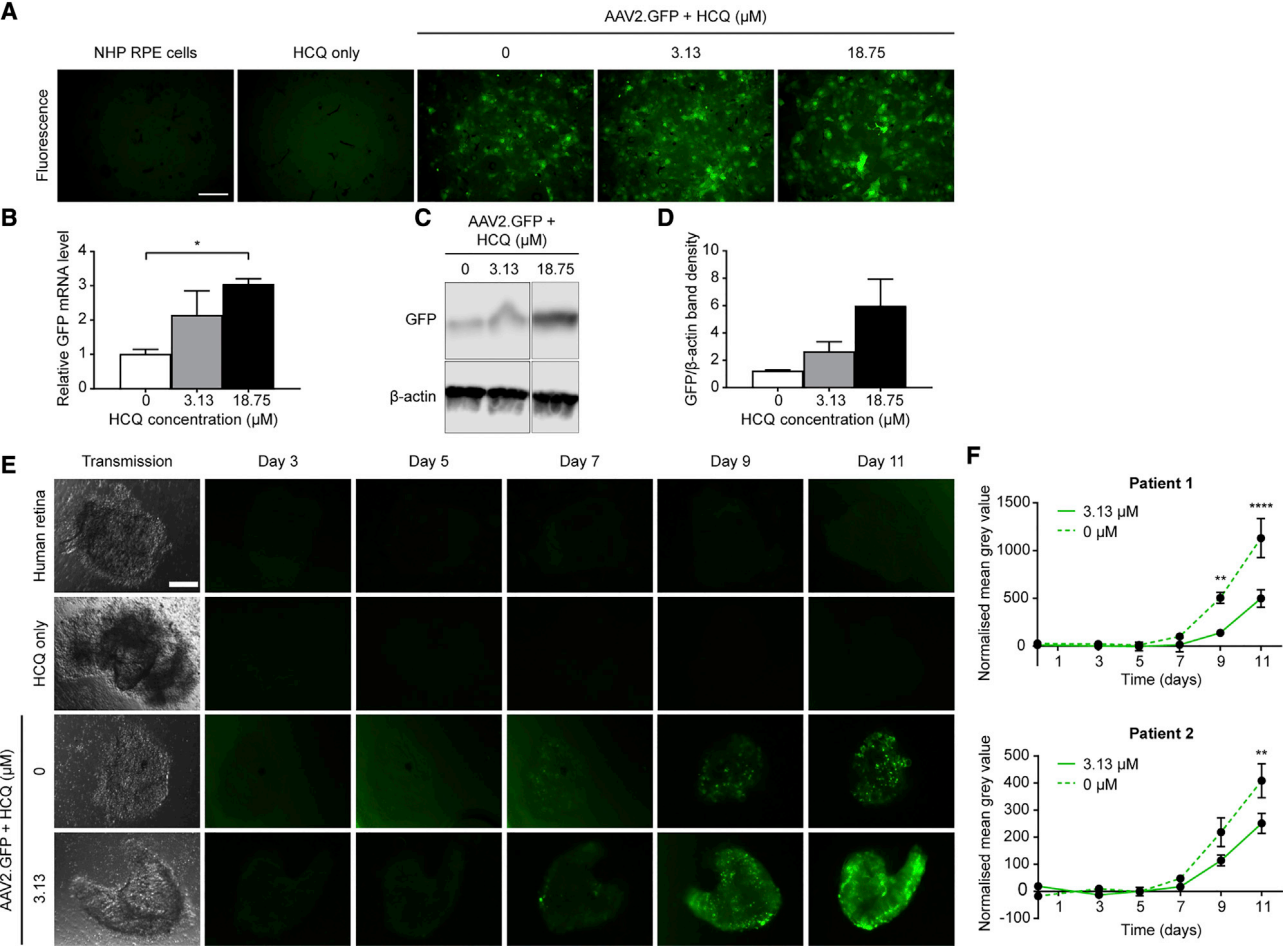
HCQ Increases AAV Transduction in Non-human Primate (NHP) Retinal Pigment Epithelium (RPE) Cells and Human Retina (A–D) Primary macaque RPE cells were treated with 0, 3.13, or 18.75 μM HCQ for 1 h prior to transduction with 2 × 10^9^ gc AAV2.GFP. (A) Representative fluorescence microscopy images acquired on day 3 post-transduction (scale bar, 200 μm). (B) Levels of *GFP* mRNA in AAV- and HCQ-treated RPE cells were quantified using qRT-PCR on day 3 post-transduction, and they are expressed as mean fold change relative to cells treated with AAV only (±SEM, n = 3). *p ≤ 0.05 (one-way ANOVA with Dunnett’s multiple comparison test). (C) Representative western blot of GFP protein expression with β-actin used as a loading control. (D) Quantification of GFP band density normalized to β-actin. Data are presented as mean ± SEM (n = 2). (E and F) Fresh patient-derived retinal explants were treated *ex vivo* with 0 or 3.13 μM HCQ for 1 h prior to transduction with 1 × 10^9^ gc AAV2.GFP (UK research ethics approval 10/H0505/8). (E) Representative transmission microscopy image at baseline and fluorescence images acquired on alternate days up to day 11 post-transduction (scale bar, 100 μm). (F) GFP expression was estimated by calculating the mean gray value of fluorescence images from two separate patients. These were normalized to untransduced controls treated with equivalent concentrations of HCQ. Data are expressed as mean ± SEM (3 replicates/patient). **p ≤ 0.01, ****p ≤ 0.0001 (two-way repeated-measures ANOVA with Šídák’s multiple comparisons test).

To assess the applicability of HCQ augmentation of AAV transduction in retinal gene therapy, primary RPE cells were isolated from non-human primate (NHP; rhesus macaque) eyes and immediately placed into culture. The RPE cells were treated with 0, 3.13, or 18.75 μM HCQ for 1 h prior to transduction with AAV2.GFP. At 3 days post-transduction, GFP expression was visualized by fluorescence microscopy and quantified by qRT-PCR and western blot. A visible increase in GFP fluorescence could be seen in RPE cells treated with HCQ compared with AAV vector alone (Figure 3A), which correlated to an up to 3-fold increase in GFP mRNA expression in cells treated with 18.75 μM HCQ (one-way ANOVA, p = 0.0253; n = 3 eyes) (Figure 3B). A similar magnitude of increase in GFP protein expression associated with HCQ was also detected by western blot; however, this did not reach statistical significance due to limited NHP tissue availability (n = 2) (Figures 3C and 3D).

Since inherited retinal degenerations can affect both RPE and photoreceptor cells, we next tested whether the effect of HCQ on AAV transduction also applied to photoreceptors. To this end, human retinal explants were collected from patients undergoing routine clinically indicated retinectomy as part of retinal detachment repair. The retinal explants were treated with either 0 or 3.13 μM HCQ for 1 h prior to transduction with AAV2.GFP. GFP fluorescence was visualized using fluorescence microscopy on days 3, 5, 7, 9, and 11 post-transduction (Figure 3E), and it was quantified using mean gray values of the explants imaged under standardized conditions.^22^ The data from retinal tissue harvested from two (of two) patients independently showed consistent trends of increased fluorescence in the explants treated with HCQ from day 7 onward when significant GFP fluorescence became detectable. A statistically significant difference was observed with the treatment effect of HCQ over time (two-way ANOVA, patient 1 p = 0.0002; patient 2 p = 0.0092; n = 3), with a mean 2-fold increase in mean gray value at day 11 for the two patients (Figure 3F).

### Co-administration of HCQ with AAV Vector Improves Retinal Gene Therapy *In Vivo* without Detectable Toxicity

To assess the translational potential of HCQ to retinal gene therapy, we tested the effect of co-administration of HCQ with AAV vector *in vivo*. The 7-week-old female C57BL/6J mice were subretinally injected with 1 × 10^8^ genome copies (gc) of AAV2.GFP containing either 3.13 or 18.75 μM HCQ in one eye and AAV vector only in the fellow eye. The fluorescence of the mouse retina was measured at regular intervals up to 8 weeks using *in vivo* confocal scanning laser ophthalmoscopy (cSLO) at standardized detector sensitivity. Cross-sectional structure of the retina was also monitored using *in vivo* spectral domain optical coherence tomography (OCT) to look for signs of retinal toxicity.

**Figure 4:**
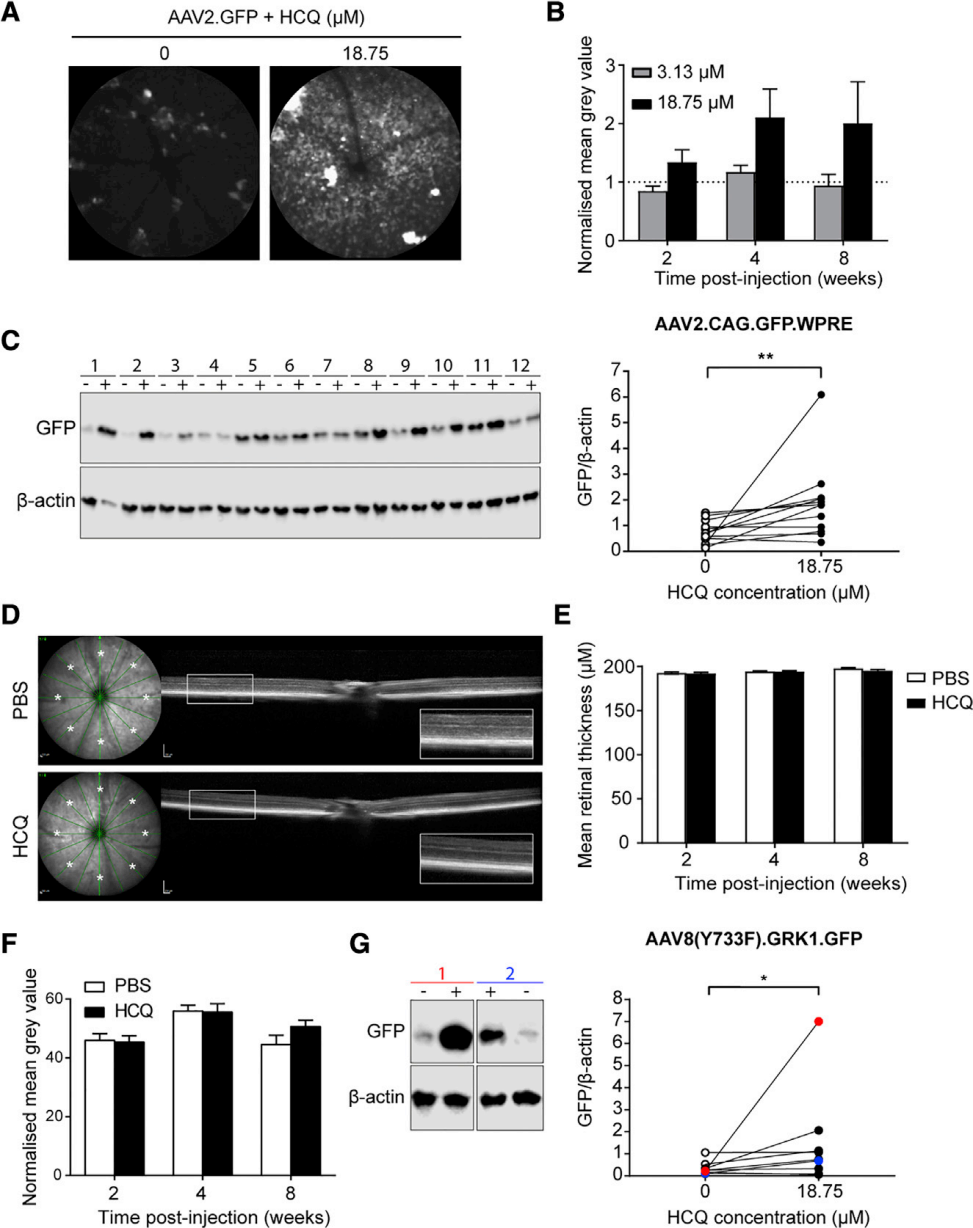
HCQ Enhances AAV Transduction of Mouse Retina *In Vivo* and Has No Detectable Effect on Retinal Architecture or Thickness (A–F) C57BL/6J mice were subretinally injected with AAV2.GFP vector alone in one eye and co-injected with either 3.13 or 18.75 μM HCQ in the fellow eye. (A) Representative *in vivo* confocal scanning laser ophthalmoscopy autofluorescence images of paired eyes at 8 weeks post-injection. (B) GFP fluorescence levels were estimated using the mean gray values of autofluorescence images. Eyes co-injected with HCQ were normalized to fellow AAV only-injected eyes at 2, 4, and 8 weeks post-transduction (±SEM; 3.13 μM, n = 5; 18.75 μM, n = 4). (C) Western blot of GFP protein levels in paired mouse retinae that received either AAV vector alone (−) or vector mixed with HCQ (18.75 μM) (+) at 8 weeks post-injection. β-actin was used as a loading control. Quantification of western blot GFP band density normalized to β-actin in paired eyes is shown (n = 12). (D) Representative spectral domain optical coherence tomography images from fellow eyes that received either PBS or 18.75 μM HCQ, showing normal retina lamellar architecture. Total retinal thickness was measured at points marked with an asterisk. (E) Mean total retinal thickness of points (*) at 2, 4, and 8 weeks post-injection (±SEM, n = 8). (F) Mean gray value of eyes injected with 18.75 μM HCQ alone normalized to paired PBS-injected eyes at 2, 4, and 8 weeks post-injection (±SEM, n = 8). (G) 129S2/SvHsd mice were subretinally injected with AAV8(Y733F).GRK1.GFP vector alone in one eye and co-injected with 18.75 μM HCQ in the paired eye. Representative western blot of GFP protein levels in paired retinae of two mice, with red and blue colors corresponding to points on the graph, is shown. Quantification of GFP band density normalized to β-actin in paired eyes is also shown (n = 9). *p ≤ 0.05, **p ≤ 0.01 (Wilcoxon matched-pairs signed rank test).

Retinal fluorescence was found to be increased in eyes that received AAV vector suspension containing 18.75 μM HCQ compared with paired eyes that received AAV vector alone (Figure 4A). No difference was seen in eyes that received AAV with 3.13 μM HCQ (Figure 4B). Although a clear trend in favor of HCQ was visible, the differences in mean retinal fluorescence based on normalized gray value comparisons did not reach statistical significance. This is likely because the method did not distinguish between the diffuse background fluorescence (which represents GFP expression) and scattered clumps of hyperfluorescence (representing autofluorescent macrophage aggregates associated with inflammation). In addition, the statistical power of the comparison was reduced by the exclusion of 4 of 13 mice (final n = 5 for 3.13 μM and n = 4 for 18.75 μM) due to unintended intravitreal injections or subretinal bleed during surgery.

To more specifically compare levels of GFP protein expression between the treatment arms, the mouse retinae were harvested for western blot analysis at the final time point (8 weeks). Eyes that received AAV with 18.75 μM HCQ showed a mean 4.6-fold increase in GFP protein level compared with paired eyes that received AAV vector alone (Wilcoxon matched-pairs signed rank test, p = 0.0034; n = 12) (Figure 4C). In terms of the safety of HCQ administration into the subretinal space, *in vivo* OCT imaging demonstrated no detectable change in lamellar retinal architecture relating to the administration of HCQ (Figure 4D). Moreover, there was no significant difference in the mean retinal thickness of eyes injected with HCQ compared to paired eyes sham injected with PBS (Figure 4E). By measuring the mean gray value of the fundus using cSLO, we demonstrated that there was no significant difference in the autofluorescence of eyes injected with PBS compared to those injected with HCQ alone, suggesting that HCQ is not autofluorescent (Figure 4F).

Next, we investigated whether HCQ was able to specifically enhance transgene expression within photoreceptors *in vivo* and whether the effect was applicable to different AAV serotypes. 4- to 5-week-old 129S2/SvHsd mice were subretinally injected with 1 × 10^8^ gc of an AAV8(Y733F) serotype vector expressing GFP under the photoreceptor-specific promoter G protein-coupled receptor kinase 1 (GRK1) (AAV8(Y773F).GRK1.GFP), with injections of AAV combined with 18.75 μM HCQ performed in the contralateral eye. Retinal GFP protein expression was found to be 5.9-fold higher in eyes injected with AAV containing 18.75 μM HCQ compared to AAV only (Wilcoxon matched-pairs signed rank test, p = 0.0391; n = 9) (Figure 4G).

### Mechanism of Action of HCQ

While HCQ is routinely used in the treatment of autoimmune diseases, its exact mechanism of action remains unclear. Several studies support the main mode of action of HCQ as via inhibition of the innate immune factors TLR9^18^, ^20^ and cGAS.^17^

**Figure 5:**
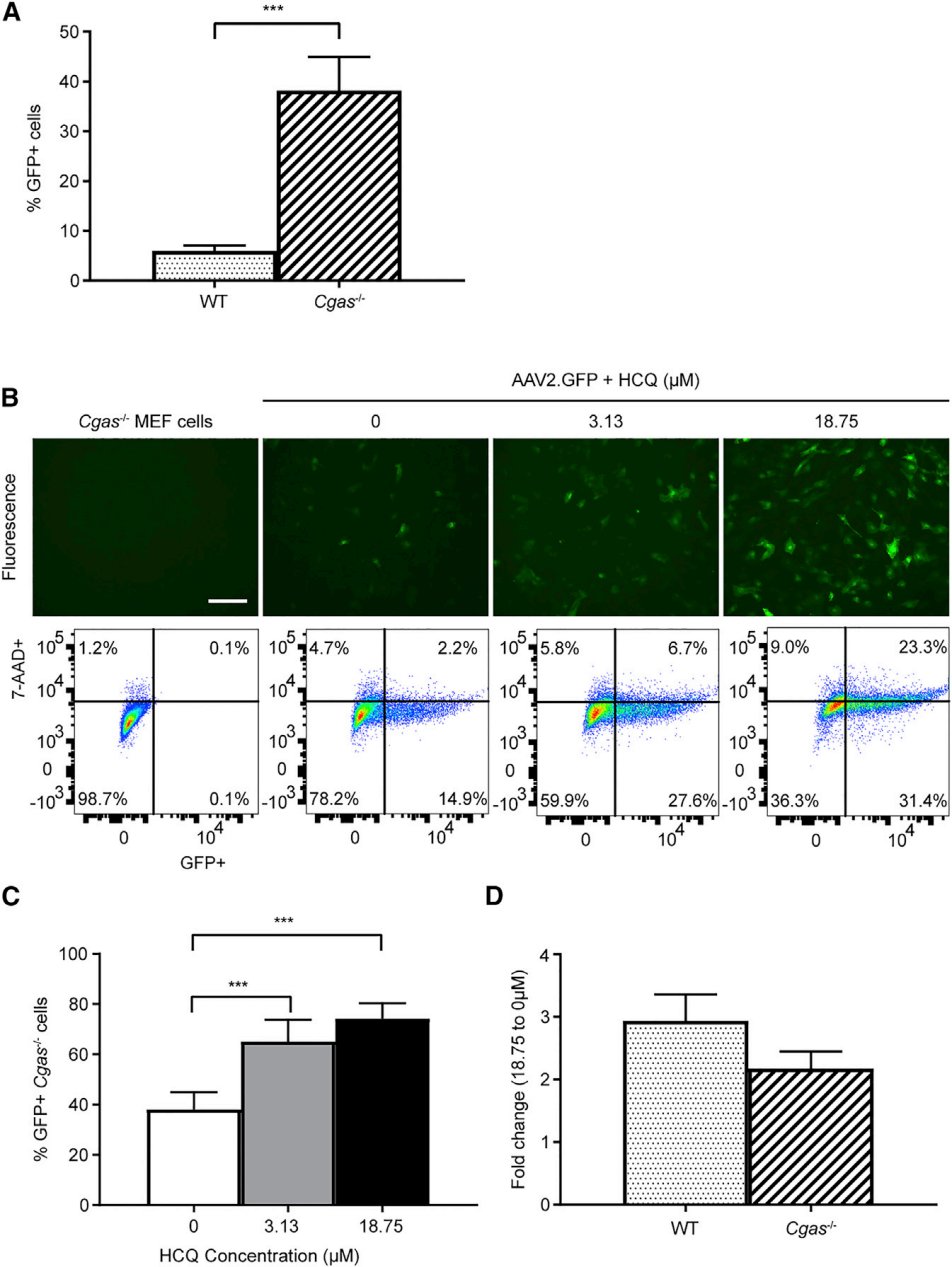
*Cgas*^−/−^ MEFs Transduce Significantly Better Than Wild-Type Cells, and HCQ Further Enhances AAV Transduction in *Cgas*^−/−^ MEFs MEFs were transduced with AAV2.GFP at an MOI of 1,000. GFP fluorescence and cell viability (7-AAD staining) were assessed on day 3 post-transduction by flow cytometry. The mean proportion of GFP-positive (GFP+) cells was expressed as a percentage of the total number of live (7-AAD-negative) cells. (A) Mean proportion of GFP+ cells in wild-type and *Cgas*^−/−^ MEFs after transduction with the same MOI (±SEM, n = 6). ***p ≤ 0.001 (paired t test). (B) Representative fluorescence images and flow cytometry plots of *Cgas*^−/−^ MEFs treated with 0, 3.13, or 18.75 μM HCQ 1 h prior to transduction (scale bar: 200 μm). (C) Mean proportion of GFP+ *Cgas*^−/−^ cells measured using flow cytometry (±SEM, n = 6). ***p ≤ 0.001 (one-way repeated-measures ANOVA with Dunnett’s multiple comparison). (D) Fold change of mean proportion of GFP+ cells treated with 18.75 μM relative to 0 μM HCQ in wild-type and *Cgas*^−/−^ MEFs. Data are presented as mean ± SEM (n = 6).

We first tested the effect of HCQ on AAV transduction in *Cgas*^−/−^ MEFs with the hypothesis that, if HCQ acted via the inhibition of cGAS, its effect would be diminished in the absence of cGAS. While we found that *Cgas*^−/−^ MEFs transduced with up to 6.4-fold greater efficacy than wild-type MEFs at the same MOI (t test, p = 0.0008; n = 6) (Figure 5A), the addition of HCQ was still able to increase the mean rate of AAV transduction from 38% with no HCQ to 65% with 3.13 μM HCQ and 74% with 18.75 μM HCQ (one-way ANOVA, p < 0.0001; n = 6) (Figures 5B and 5C). Consequently, there was no significant difference in the effects of 18.75 μM HCQ on wild-type versus *Cgas*^−/−^ MEFs (Figure 5D). This indicates that HCQ is unlikely to act exclusively via the inhibition of cGAS.

**Figure 6:**
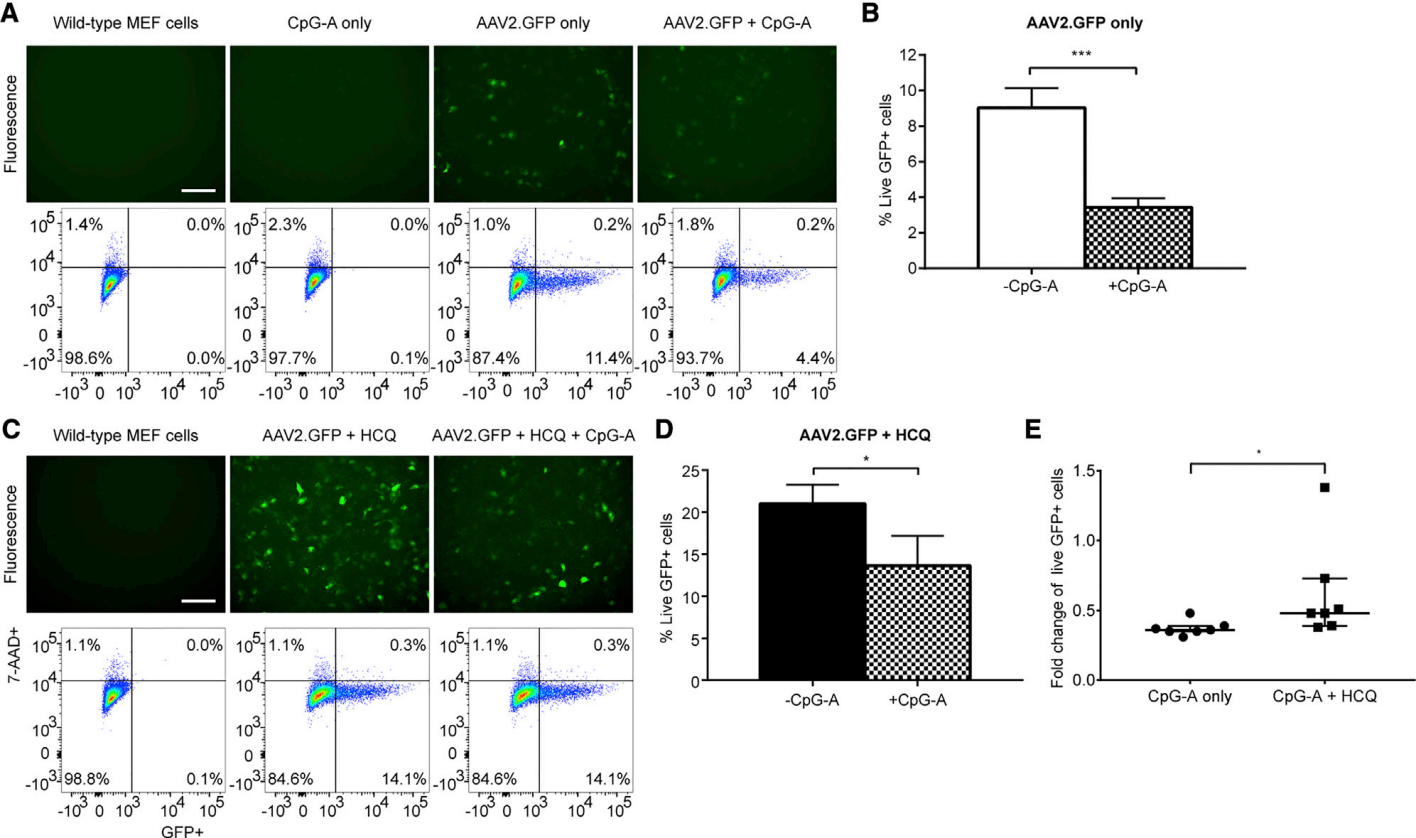
The Agonistic TLR9 Oligodeoxynucleotide CpG-A Decreases AAV Transduction in Wild-Type MEFs and Has a Significantly Reduced Effect in HCQ-Treated Cells Wild-type MEFs were treated with or without CpG-A (754.6 μM) 30 min prior to transduction with AAV2.GFP at an MOI of 1,000. GFP fluorescence and cell viability (7-AAD staining) were assessed on day 3 post-transduction by flow cytometry. (A) Representative fluorescence images and flow cytometry plots (scale bar, 200 μm). (B) The mean proportion of GFP-positive (GFP+) cells expressed as a percentage of the total number of live (7-AAD-negative) cells (±SEM, n = 7). ***p ≤ 0.001 (paired t test). (C and D) Wild-type MEFs were treated with 18.75 μM HCQ 1 h prior to transduction with AAV2.GFP, followed by treatment with or without CpG-A 30 min prior to transduction. (C) Representative fluorescence images and flow cytometry plots (scale bar, 200 μm). (D) The mean proportion of GFP+ cells (±SEM, n = 7). *p ≤ 0.05 (paired t test). (E) The fold change of transduced live GFP+ cells treated with CpG-A relative to cells with no CpG-A treatment. Cells were either treated with CpG-A alone or with CpG-A and HCQ (median ± interquartile range, n = 7). *p ≤ 0.05 (Wilcoxon matched-pairs signed rank test).

Next we explored the role of TLR9 in AAV transduction with the use of the agonistic TLR9 oligodeoxynucleotide CpG-A,^23^ which contains unmethlyated CpG sequences known to stimulate TLR9 activity.^24^ Cells were treated with CpG-A for 30 min prior to AAV2.GFP transduction with or without HCQ, and GFP fluorescence was quantified using flow cytometry 3 days post-transduction. CpG-A treatment significantly reduced the mean number of GFP-fluorescing cells by 2.7-fold relative to untreated cells (Figures 6A and 6B) (paired t test, p = 0.0001; n = 7) and by 1.9-fold in HCQ-treated cells (Figures 6C and 6D) (p = 0.0348; n = 7). Therefore, the inhibitory effect of CpG-A on AAV transduction was significantly reduced by the presence of HCQ (Figure 6E) (Wilcoxon matched-pairs signed rank test, p = 0.0156; n = 7).

To assess the effect of HCQ on viral entry, we compared the intracellular AAV genome loads up to 1 h post-transduction between HCQ-treated and untreated wild-type MEFs. No significant difference in overall intracellular AAV genome copy numbers was detected by qPCR between HCQ-treated and untreated cells (Figure S4). Further analysis of subcellular localization of AAV genomes was conducted by cell fractionation followed by qPCR in HCQ-treated versus untreated wild-type MEFs 24 h post-transduction. The presence of HCQ was found to alter the subcellular distribution of AAV with a trend toward increased nuclear localization (two-way ANOVA, p = 0.0356; n = 3) (Figure S5B). However, the effect did not reach statistical significance upon multiple comparison testing due to the high level of variation in the qPCR assay. Successful separation of cytosolic versus nuclear fractions was confirmed using the cytosolic marker α-tubulin and nuclear marker histone H3 (Figure S5A). Genomic glyceraldehyde 3-phosphate dehydrogenase (GAPDH) DNA was used as a marker for nuclear DNA (Figure S5C).

## Discussion

Despite AAV vectors being considered to have low immunogenicity, particularly when administered into the subretinal space, our data suggest that AAV transduction is associated with the activation of intracellular innate immunity as well as downstream proinflammatory cytokines independent of surgical trauma. The induction of *Tnf-α*, *IFN-γ*, and type I interferon responses could lead to the breakdown of the blood-retina barrier and recruitment of adaptive immunity.^25^, ^26^, ^27^ This occurrence would ultimately cause localized tissue inflammation and reduced therapeutic efficacy, and it may underlie the cases of intraocular inflammation encountered in retinal gene therapy trials.^14^, ^15^, ^16^

The upregulation of innate immune mediators detected on day 7 post-subretinal gene therapy *in vivo* does not rule out the presence of background levels of pre-existing surveillance innate sensors. Active AAV particles may be present within the subretinal space for a prolonged period (e.g., days) during which repeated waves of viral entry into the host cells may occur. The signaling molecule generated by activated cGAS, cyclic GMP-AMP (cGAMP) has been shown to traverse intercellular junctions and cause field effects, which could account for the delayed peaking of innate immune responses.^28^ In addition, significant GFP mRNA expression was not seen *in vivo* until 7 days post-injection, which corresponds to the induction of the innate immune genes and may also suggest a delay in the uptake of AAV from the subretinal space. Retinal microglia are resident macrophages that play an important role in immune defense in the eye, and they are capable of detecting viruses and secreting inflammatory cytokines to amplify an inflammatory response.^29^ As these cells are capable of mounting an anti-viral response through the activation of cGAS^30^ and TLR9,^31^ we cannot rule out the possibilities that resident microglia may have been responsible for the upregulation of the innate immune factors and that the delay could represent the activation of resident microglia or recruitment of lymphocytes.

HCQ is an approved antimalarial drug with a well-established safety profile. It is a putative inhibitor of innate immunity, consistent with known efficacy in the treatments of autoimmune diseases such as systemic lupus erythematosus and rheumatoid arthritis.^19^, ^32^ A recent *in silico* analysis suggested that HCQ can disrupt dsDNA binding and the activation of cGAS.^17^ However, we found that HCQ did not discriminate in its ability to improve AAV transduction between wild-type and *Cgas*^−/−^ MEFs, which suggests that HCQ is unlikely to act exclusively through cGAS. Also, although *Cgas*^−/−^ MEFs transduce with 6.4-fold greater efficiency than wild-type MEFs, this does not necessarily imply that cGAS is the sole limiting factor for AAV transduction, given that the absence of cGAS is likely to affect the expression of other intracellular innate immune genes.

Additional studies have proposed that HCQ may act through the inhibition of TLR9. One group suggested that HCQ may bind to DNA and cause conformational changes that prevent the activation of TLR9,^18^ while others have suggested that the effect of HCQ on endosomal pH can indirectly inhibit TLR9, which is localized within endosomes and activated by an acidic environment.^20^ We found that treatment of wild-type MEFs with a TLR9-activating oligonucleotide (CpG-A) caused a 2.7-fold reduction in transgene expression, suggesting that the stimulation of TLR9-mediated innate immunity can lead to reduced AAV transduction. HCQ significantly, but incompletely, abrogated the inhibitory effect of CpG-A on AAV transduction, suggesting that HCQ may be an antagonist of TLR9. If HCQ prevents the activation of TLR9 by causing conformational changes in DNA,^18^ it is possible that the partial response seen may be a result of the stoichiometric ratio between HCQ and CpG-A, such that some unbound CpG-A was able to adversely affect AAV transduction.

However, the possibility of HCQ acting via alternative targets cannot be ruled out. Our viral entry assay did not show any notable difference in AAV viral loads soon after transduction, possibly indicating that HCQ does not act by decreasing degradation or by increasing AAV binding or entry into the target cells. Investigation into the subcellular localization of AAV revealed a strong trend toward shifting from the cytosol to the nucleus upon HCQ treatment. This observation is potentially consistent with evasion of intracellular innate immunity, and it could account for the increased transgene expression seen following HCQ treatment. Further work is required to clarify the molecular mechanism of action of HCQ, and it seems likely that the proposed interactions of HCQ are not mutually exclusive, as the drug may have multimodal effects.

It should be noted that a previous study using a much higher concentration of CQ (at 100 μM, compared with up to 12 μM in this study) showed a reduction in AAV2 transduction in a human hepatocellular carcinoma cell line.^33^ The difference may be the result of the CQ concentrations used, e.g., greater lysosomal acidification at the much higher drug concentration may inhibit lysosomal escape of AAV particles. However, since CQ is known to be associated with a far greater chance of retinal toxicity than HCQ at therapeutic concentrations, significant differences in their mechanisms of action and effects on AAV transduction may exist but are outside the scope of this translational study.

Our data demonstrate that HCQ is able to increase GFP transgene expression following AAV transduction *in vitro*, *ex vivo*, and *in vivo* across a range of species. The data also suggest that HCQ can improve AAV transduction of both RPE and photoreceptors, two key cell targets for retinal gene therapy. Subretinal injection of HCQ together with an AAV vector led to increased transgene expression, which was sustained up to 8 weeks. This effect was seen with both an AAV2 and AAV8(Y733F) vector, suggesting HCQ has an effect across multiple serotypes of AAV commonly used in retinal gene therapy. Furthermore, we have demonstrated HCQ to work with vectors containing both ubiquitous and photoreceptor-specific promoters, suggesting that adjunctive use of HCQ is capable of increasing transgene expression levels in photoreceptor cells, which are the primary target for many retinal degenerations.

While long-term high-dose systemic HCQ therapy is known to carry a cumulative risk of retinopathies,^34^ the risk of retinal toxicity associated with locally administered HCQ was previously unknown. Our *in vivo* data indicate that the delivery of a single pulse of HCQ into the subretinal space was not associated with any retinal structural change, including preservation of the ellipsoid zone on the OCT within treated areas, which represents organized photoreceptor inner and outer segments up to 8 weeks post-injection. One of the limitations of the study is that, while HCQ enhanced retinal gene therapy in the healthy retina of wild-type mice without any sign of toxicity, it remains to be seen whether the effects would be the same in the degenerate retina of mouse models of retinitis pigmentosa, which would provide support for future clinical application.

The proportion of retinal cells that successfully acquire and maintain transgene expression determines the clinical efficacy of gene therapy in neurodegenerative diseases. For X-linked and autosomal recessive retinitis pigmentosa, which account for approximately 40% of patients with identified mutations, achieving over 50% photoreceptor transduction may be seen as a threshold for halting disease progression, since female carriers of X-linked mutations are usually minimally affected (e.g., choroideremia, X-linked retinitis pigmentosa associated with mutations in *RPGR* or *RP2*). Therefore, a 2-fold increase in transgene expression with the adjunctive use of HCQ has the potential to push the therapeutic effect beyond the threshold required to halt disease progression. Alternatively, co-administration of HCQ with the AAV vector could enable a lower vector dose to be used to achieve a given therapeutic effect, thus reducing the risk of inflammation and improving safety for the patient. The approach is broadly applicable and has the potential to augment the effects of existing gene therapies under investigation, without the need to modify the clinical grade vector, an important practical consideration given the prohibitive costs of developing gene therapies for orphan diseases.

## Materials and Methods

### Vector Design

The vector pAAV2.CAG.GFP.WPRE.pA contained a GFP transgene under control of the ubiquitous CAG promoter with a Woodchuck hepatitis virus post-transcriptional regulatory element (WPRE) enhancer. The vector pAAV8(Y733F).GRK1.GFP.pA contained the reporter GFP under control of a photoreceptor-specific promoter GRK1. Both vectors were single-stranded AAVs.

### AAV Production

HEK293T cells were grown in HYPERFlasks (Scientific Laboratory Supplies) and transfected with a total of 500 μg endotoxin-free plasmid using polyethylenimine (Sigma-Aldrich). The plasmid pCAG.GFP.WPRE.pA was co-transfected with pDG (PlasmidFactory) to package into recombinant AAV serotype 2, and pGRK1.GFP.pA was co-transfected with pRepCap and pHelper (pDeltaAdF6) for packaging into AAV serotype 8 Y733F. Then 3 days post-transfection, the cells were harvested, lysed, and treated with nuclease. The AAV population was isolated from the cell lysates by ultracentrifugation with an iodixanol gradient and purified using Amicon Ultra-15 100K filter units (Merck Millipore). The final preparations were washed and collected in sterile PBS. The purity of the AAV preparations were determined by SDS-PAGE analysis, followed by staining with EZBlue (Sigma-Aldrich) (Figure S1), and the titers were determined by qPCR. Lot 1 of AAV2.CAG.GFP.WPRE was used for Figure 1, lot 3 was used for Figure S5, and lot 2 was used for all other experiments.

### Mice

Wild-type C57BL/6J mice (Charles River Laboratories) were maintained by the Biomedical Science Division, University of Oxford, UK. Mice were housed in a 12 h light-dark cycle, with food and water available *ad libitum*. All procedures were performed under general anesthesia. All animal procedures were undertaken in accordance with the Association for Research in Vision and Ophthalmology guidelines for the humane use of laboratory animals in ophthalmic research, and they were approved by local and national ethical and legal authorities.

### Subretinal AAV Vector-Mediated Gene Therapy

For assessment of the activation of innate immune factors, 4-week-old female C57BL/6J mice were subretinally injected with 1.5 μL (1 × 10^9^ gc) of the vector pAAV2.CAG.GFP.WPRE.pA (AAV2.GFP)^35^ in the left eye and sham injected with PBS in the right eye (n = 3/time point). For GFP expression analysis, mice were injected with 1 × 10^8^ gc (n = 7/time point). Two mice (four for GFP expression analysis) were left uninjected to determine baseline gene expression. Retinas were harvested at baseline and on days 3, 7, and 15 post-injection.

For assessment of the effect of HCQ *in vivo*, 7- to 8-week-old female C57BL/6J mice were subretinally injected with 1.2 μL (1.2 × 10^8^ gc) AAV2.GFP, with injections of a suspension of AAV2.GFP with either 3.13 μM (n = 7) or 18.75 μM (n = 6) HCQ performed in the contralateral eye. An additional nine mice were injected with 18.75 μM HCQ and AAV in one eye and AAV only in the paired eye to supplement the cohort for western blot analysis (n = 13). To assess the toxicity and autofluorescence of HCQ, 7- to 8-week-old female C57BL/6J mice were subretinally injected with 1.2 μL 18.75 μM HCQ, with sham injections of PBS undertaken in the paired eye (n = 12).

The 4- to 5-week-old 129S2/SvHsd mice were subretinally injected with 1 μL (1 × 10^8^ gc) AAV8(Y733F).GRK1.GFP, with injections of AAV8(Y733F).GRK1.GFP with 18.75 μM HCQ performed in the paired eye (n = 9).

### Cell and Tissue Cultures

MEF cells were cultured in DMEM supplemented with L-glutamine (2 mM), penicillin (100 U/mL), streptomycin (100 μg/mL), and 10% fetal bovine serum. Cells were maintained at 37°C in a humidified 5% CO_2_ environment. Wild-type and *Cgas*^−/−^ MEFs were transduced with AAV2.GFP at an MOI of 1,000. When appropriate, cells were incubated in media with HCQ or CQ for 1 h prior to transduction. The cells were cultured in HCQ- or CQ-containing media for 3 days post-transduction, at which point fluorescence and transmission images were acquired and cells were harvested for flow cytometry. TLR9 was activated in wild-type MEFs by pre-treatment with 754.6 μM CpG-A (5′-GGGGGACGATCGTCGGGGGG-3′) for 30 min prior to transduction with AAV2.GFP. CpG-A was phosphorothioated at each base (Integrated DNA Technologies). For assessing viral entry, cell pellets were washed four times with PBS and harvested for DNA extraction 0, 10, 30, and 60 min post-transduction. For assessing subcellular localization, cell pellets were washed four times with PBS, and the cytosolic and nuclear fractions were isolated using the Cell Fractionation Kit (ab109719, Abcam), according to the manufacturer’s instructions.

Primary NHP RPE cells were derived from rhesus macaque (*M. mullata*) eyes collected post-mortem from animals euthanized for other purposes, provided by the MRC Centre for Macaques (Porton Down, UK). After enucleation, the cornea and lens were removed under direct visualization with a surgical microscope. Radial incisions were made toward the posterior pole to flatten the eyecup. The retina was removed by blunt dissection, and the remaining eyecup was placed in media and stored on ice until the RPE cells were removed. RPE cells were detached using 2.5% trypsin, and they were cultured in advanced DMEM supplemented with L-glutamine (2 mM), penicillin (100 U/mL), streptomycin (100 μg/mL), and 10% fetal bovine serum. Cells were maintained at 37°C in a humidified 5% CO_2_ environment. The RPE cells were incubated in media with 0, 3.13, or 18.75 μM HCQ for 1 h prior to transduction with AAV2.GFP at 2 × 10^9^ gc/well of a 12-well plate (n = 3). The cells were cultured in HCQ-containing media for 3 days post-transduction, at which point fluorescence and transmission images were acquired and the cells were harvested for RNA and protein extraction using buffer RLT (QIAGEN).

Fresh human retinal explants were collected from two patients with informed consent during routine retinal detachment surgery where a retinectomy was clinically indicated, with approval by the UK National Research Ethics Committee (10/H0505/8). Small retinal fragments approximately 500 μm in diameter were obtained using a 23G cutter of a surgical vitrectomy system (Constellation Vision System, Alcon). Within 1 h of tissue collection, individual retinal fragments were transferred using a Pasteur pipette into organotypic culture inserts (Falcon, Corning) within 24-well plates. The retinal explants were cultured in 500 μL Neurobasal-A medium supplemented with L-glutamine (0.8 mM), penicillin (100 U/mL), streptomycin (100 μg/mL), 2% B-27 Supplement, and 1% N-2 Supplement (Thermo Fisher Scientific). Samples were maintained at 34°C in a humidified 5% CO_2_ environment. After 24 h, the fragments were transferred to culture media containing either 0 or 3.13 μM HCQ for 1 h prior to transduction with AA2.GFP at 1 × 10^9^ gc/well (n = 3/patient).

Fluorescence and transmission microscopy images were obtained on alternate days up to day 11 under standardized conditions. The culture media were replaced on alternate days with fresh media containing the same concentration of HCQ. To measure the mean gray value of an explant, as a surrogate for GFP fluorescence, the area of unfolded retina was traced on the transmission image and overlaid onto the fluorescence image in ImageJ (NIH, USA).^22^, ^36^ To account for background autofluorescence of the well, the mean gray value of a 2.54-mm band around the retinal tissue was subtracted from the mean gray value of the tissue. Normalized gray value was then calculated by subtracting the mean gray value of untransduced retinal tissue (treated with the same HCQ concentration) to account for any background autofluorescence of the tissue and drug.

### Flow Cytometry

Dissociated cells were fixed in 4% methanol-free paraformaldehyde in PBS for 15 min at room temperature. The fixative was replaced with 1% BSA in PBS and samples were stored on ice until assessment. Cells were incubated with the cell viability dye 7-AAD for 5 min in the dark immediately prior to flow cytometry analysis using a BD LSRFortessa. Fluorescent light was measured using a blue laser at the band-pass filter 695 nm/40 mW and 530 nm/30 mW to detect the fluorochromes of 7-AAD and GFP, respectively. Cells expressing GFP were gated for using untransduced controls, and dead (7-AAD-positive) cells were excluded using a dead cell population control prepared by heating the samples to 60°C for 20 min.

### qRT-PCR

Total RNA was extracted from mouse retinae and NHP RPE cells using the RNeasy mini kit (QIAGEN). An additional DNA digestion step was included for the extraction from NHP RPE cells using the RNase-free DNase kit (QIAGEN). Buffer RLT lysates were kept from the NHP RPE cells for protein extraction according to the manufacturer’s instructions. cDNA was synthesized with an oligonucleotide deoxythymine (dT) primer using the Superscript III Kit (Invitrogen). qPCR of the mouse retinal cDNA samples was undertaken using iTaq Universal SYBR Green Mastermix (Bio-Rad) for *Apobec3*, *Apobec2*, and *Aicda*. qPCR of the other innate immune genes (*Tlr9*, *Mb21d1*, *Ddx58*, *Sting*, *Trim21*, *Ifn-γ*, *Tnfα*, *Cxcl10*, and *Isg15*) was undertaken using TaqMan probes (Thermo Fisher Scientific) and TaqMan Fast Universal PCR Master Mix (Applied Biosciences). qPCR of *GFP* expression in mouse retinae and NHP RPE cells was performed using specific TaqMan probes and Fast Universal PCR Master Mix. Gene expression of all genes was normalized to *β-actin* and analyzed according to the 2^(−ΔΔCt)^ method.

DNA was extracted using the QIAamp DNA Mini Kit (QIAGEN) from wild-type MEFs; an additional RNase digest was performed on the cell fractionation samples. qPCR was performed using *GFP* and *Gapdh* genomic DNA copy number TaqMan probes and Fast Universal PCR Master Mix.

### *In Vivo* Retinal Imaging

cSLO imaging was performed on anesthetized C57BL/6J mice at 2, 4, and 8 weeks post-subretinal gene therapy using a 55° lens (Spectralis HRA, Heidelberg Engineering, Heidelberg, Germany), with a real-time average process of 25 frames and a standardized signal detector sensitivity of 70. Fundus autofluorescence imaging was measured within a ring section with a radius between 350 and 860 pixels from the optic disc, using the ImageJ software (NIH).^36^ Animals were also subject to wide-field OCT (Spectralis HRA, Heidelberg Engineering) 2, 4, and 8 weeks post-injection. Mice were scanned using a 55° lens, and 8 radial sections were taken with a real-time average process of 25 frames. The total retinal thickness was manually measured 3,700 nm from the optic disc at alternate radial sections using a calliper. Mice with unintended intravitreal injections, significant subretinal bleeding, or opacities were excluded from analysis. For the AAV-injected mice, the number included for final SLO and OCT analysis was n = 5 for those injected with 3.13 μM HCQ and n = 4 for mice injected with 18.75 μM HCQ, and for western blot analysis the number included was n = 12. For mice injected with HCQ only in the toxicity study, the final cohort size was n = 8.

### Western Blot Analysis

Mouse retinae were lysed in radioimmunoprecipitation (RIPA) buffer supplemented with protease inhibitors (PIs) and homogenized using a hand-held homogenizer. NHP RPE protein lysates extracted from buffer RLT (QIAGEN) were resuspended in 1% SDS. For confirmation of the isolation of cytosolic versus nuclear fractions, PI was added to Buffer A (ab109719, Abcam) before cell fractionation. Cytosolic proteins were lysed in Buffer A and nuclear proteins were lysed in RIPA buffer supplemented with PI.

Protein concentration was measured using a bicinchoninic assay (Thermo Fisher Scientific). Protein samples were separated using SDS-PAGE on a 10% or 4%–20% (for Figure S5A) Criterion TGX Precast gel (Bio-Rad), and they were transferred to a polyvinylidene difluoride membrane using the TransBlot Turbo system (Bio-Rad). Membranes were blocked in 3% BSA in PBS + 0.1% Tween-20 prior to incubation for 1 h with anti-GFP (ab32146, Abcam) or anti-β-actin (AM4302, Thermo Fisher Scientific) monoclonal primary antibodies. Cell fractionation membranes were blocked in 5% donkey serum in Tris-buffered saline + 0.1% Tween-20 prior to incubation with anti-histone H3 (ab7291, Abcam) and anti-α-tubulin (ab1791, Abcam) antibodies. After washing in buffer, GFP- and β-actin-probed membranes were incubated with a corresponding horseradish peroxidase-conjugated secondary antibody for 30 min (Abcam) and developed using Clarity ECL (Bio-Rad). Histone H3- and α-tubulin-probed membranes were incubated with corresponding IRDye secondary antibodies (LI-COR Biosciences). Membranes were detected using the Odyssey Imaging System (LI-COR Biosciences). Protein band densities were quantified using the ImageStudio Lite software (LI-COR Biosciences).

### Statistical Analysis

All statistical analysis was performed on GraphPad Prism 7.00. Datasets that were normally distributed were analyzed using a parametric test (t test or ANOVA). Data were assessed for equal variance using the Brown-Forsythe test. Data that were skewed or had an unequal variance were analyzed using a non-parametric test (Wilcoxon signed rank test). Multiple comparison corrections were performed using a Dunnett or Šídák’s test for a one- or two-way ANOVA, respectively. Paired data were handled accordingly. All statistical tests were performed using an alpha level of 0.05 and using two-tailed testing. The n and p values are indicated in the figure legends where appropriate. Data are presented as mean ± SEM.

## Author Contributions

Conceptualization, L.C.C., A.R.B., C.R., R.E.M., and K.X.; Methodology, L.C.C., A.R.B., S.L.C., M.I.P., M.E.M., H.F., and C.R.; Formal Analysis, L.C.C., S.L.C., and H.F.; Investigation, L.C.C., A.R.B., S.L.C., M.I.P., M.E.M., and H.F.; Resources, C.R.; Writing – Original Draft, L.C.C.; Writing – Review & Editing, L.C.C., A.R.B., M.I.P., M.E.M., R.E.M., and K.X.; Supervision, R.E.M. and K.X.; Funding Acquisition, K.X.

## Conflicts of Interest

K.X., L.C.C., R.E.M., A.R.B., and M.I.P. have filed a patent on behalf of the University of Oxford relating to the use of hydroxychloroquine as an adjunct for gene therapy (PCT/EP2019/053193). R.E.M. is a scientific co-founder of Nightstar Therapeutics Inc., a gene therapy company established by the University of Oxford and originally funded by the Wellcome Trust through Syncona Partners Ltd.

## Supplemental Information

Document S1. Figures S1–S5

Document S2. Article plus Supplemental Information
