# Enhancement of Adeno-Associated Virus-Mediated Gene Therapy Using Hydroxychloroquine in Murine and Human Tissues

**DOI:** 10.1016/j.omtm.2023.01.001

**Published:** 2023-02-13

**Authors:** Laurel C. Chandler, Alun R. Barnard, Sarah L. Caddy, Maria I. Patrício, Michelle E. McClements, Howell Fu, Cristina Rada, Robert E. MacLaren, Kanmin Xue

## Main text

(Molecular Therapy: Methods & Clinical Development *14*, 77–89; September 2019)

In Figure 6C of the original published version of this article, the flow cytometry plot in the condition “AAV2.GFP + HCQpG-A” was inadvertantly duplicated from “AAV2.GFP + HCQ.” The duplicated plot has been replaced with the correct version. This minor editorial error did not affect the results or conclusions of the manuscript.

The authors apologize for the error.

**Figure:**
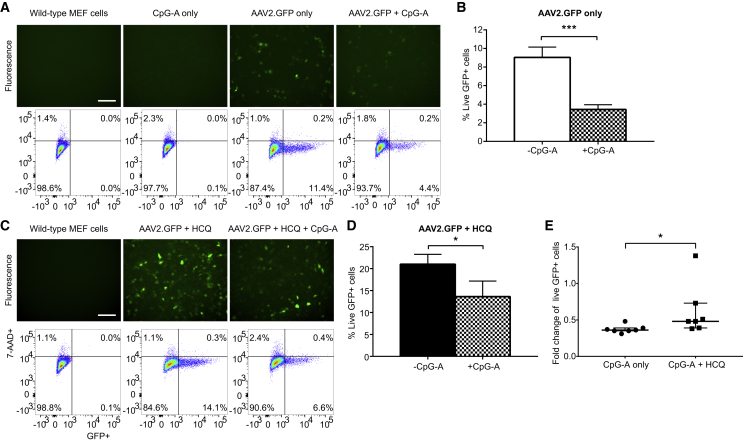
Figure 6. The Agonistic TLR9 Oligodeoxynucleotide CpG-A Decreases AAV Transduction in Wild-Type MEFs and Has a Significantly Reduced Effect in HCQ-Treated Cells (corrected)

