# Supplementary material for: Enhancement of Adeno-Associated Virus-Mediated Gene Therapy Using Hydroxychloroquine in Murine and Human Tissues
Source: Mol Ther Methods Clin Dev. 2019 Jun 4;14:77–89. doi: 10.1016/j.omtm.2019.05.012 (PMC6606965; doi:10.1016/j.omtm.2019.05.012)
Supplement: Document S1. Figures S1–S5 [file mmc1.pdf]

OMTM, Volume *14*

Supplemental Information

Enhancement of Adeno-Associated Virus-Mediated Gene Therapy Using Hydroxychloroquine in Murine and Human Tissues

Laurel C. Chandler, Alun R. Barnard, Sarah L. Caddy, Maria I. Patrício, Michelle E. McClements, Howell Fu, Cristina Rada, Robert E. MacLaren, and Kanmin Xue

Supplemental figures

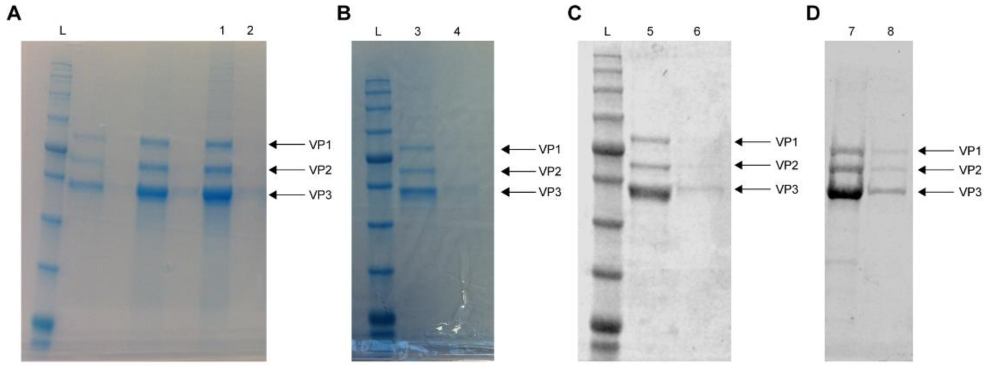

Fig S1. EZBlue stained SDS-PAGE of AAV vectors to assess purity. Image of the purified preparation of AAV2.GFP (**A**) lot #1, (**B**) lot #2 and (**C**) lot #3. (**D**) Purified preparation of AAV8(Y733F).GRK1.GFP, image taken on Odyssey Imaging System (LI-COR Biosciences). The presence of AAV virion protein 1 (VP1), VP2 and VP3 are indicated. Lanes 1, 3 & 5: AAV2.GFP concentrated sample; lanes 2, 4 & 6: AAV2.GFP wash; lane 7: concentrated AAV8(Y733F).GRK1.GFP; lane 8: AAV8(Y733F).GRK1.GFP wash; L: protein ladder.

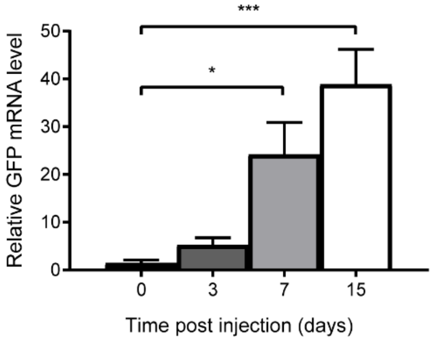

Fig S2. GFP expression following AAV2.GFP subretinal injection *in vivo*. Wild-type C57BL/6 mice were subretinally injected with AAV2.GFP ($1.2x10^8$ gc). RNA was extracted from the mouse retina at baseline (n=5) and on day 3 (n=6), 7 (n=6) and 15 (n=7) post-injection. GFP expression was quantified using RT-qPCR. Relative expression was calculated as a mean fold change (±SEM) relative to the mean of uninjected baseline controls. **p*≤0.05 and ****p*≤0.001 (one-way ANOVA with Dunn's multiple comparison test).

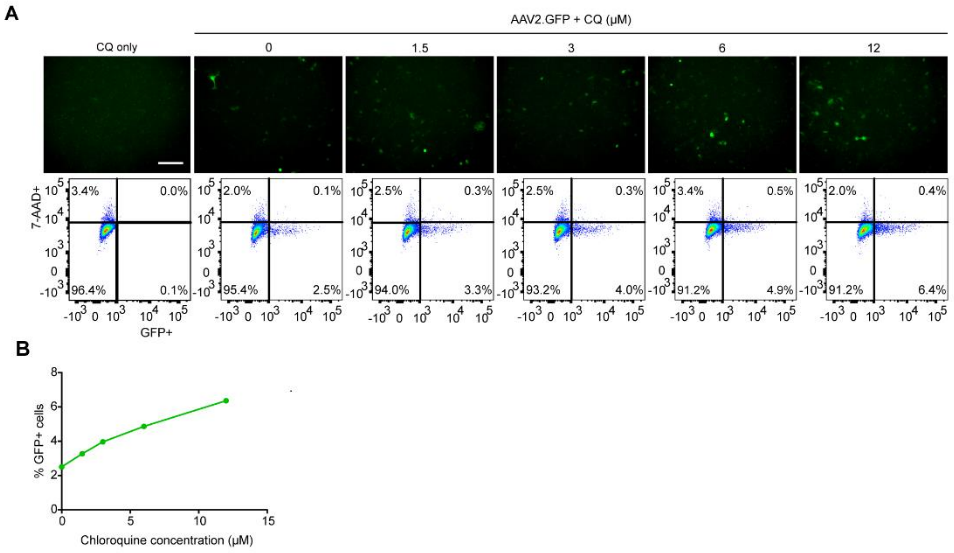

Fig S3. Chloroquine (CQ) increases AAV transduction in MEFs. Wild-type MEFs were pre-treated with 0, 1.5, 3, 6 or 12 μM of CQ for 1h prior to transduction with AAV2.GFP at a MOI of 1000. (**A**) Representative fluorescence microscopy images acquired at day 3 post-transduction (scale bar: 200 μm) shown alongside flow cytometry analyses gated for GFP fluorescence and the cell viability marker, 7-AAD. (**B**) Proportion of GFP positive (GFP+) cells expressed as a percentage of the total number of live (7-AAD negative) cells at day 3 (n=1).

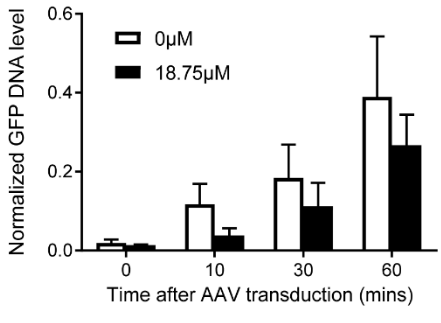

Fig S4. Effect of HCQ on intracellular AAV genome load post-transduction. Wild-type MEFs were pre-treated with HCQ for 1h prior to transduction with AAV2.GFP at a MOI of 1000. Cells treated with 0 or 18.75 μM HCQ were harvested at 0, 10, 30 and 60 mins post-transduction. DNA was extracted and *GFP* DNA copy numbers were quantified using qPCR. *GFP* DNA was normalized to *Gapdh* genomic DNA levels (±SEM, n=3).

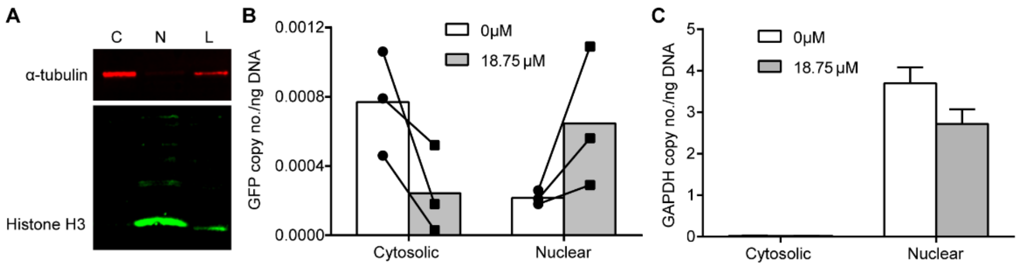

Fig S5. HCQ influences the subcellular distribution of AAV viral genomes. Wild-type MEFs were pre-treated with 0 or 18.75 μM HCQ for 1h prior to transduction with AAV2.GFP at a MOI of 1000. Cells were harvested 24h post-transduction and DNA was extracted. (**A**) Western blot of α-tubulin and histone H3 protein expression in cytosolic [C] and nuclear [N] fractions, and unfractionated whole cell lysate [L]. (**B**) Paired data of 0 or 18.75 μM HCQ treated samples plotted onto mean *GFP* DNA copy numbers per ng of DNA quantified using qPCR (n=3). (**C**) *Gapdh* genomic DNA levels (±SEM, n=3).
